# Severity-Dependent Peripheral Circulatory Responses to Neuroprosthetic Functional Electrical Stimulation in Chronic Stroke: A Four-Week Time Course Study

**DOI:** 10.7759/cureus.94441

**Published:** 2025-10-13

**Authors:** Masaya Tanabe, Akira Kimura

**Affiliations:** 1 Physical Therapy, Gunma Paz University, Takasaki, JPN; 2 Graduate Shcool of Health Sciences, Gunma Paz University, Takasaki, JPN

**Keywords:** blood flow estimation, chronic stroke, functional electrical stimulation, peripheral circulation, stroke

## Abstract

Background and objective

Chronic hemiplegia after stroke often results in disuse of the paretic limb, impaired peripheral circulation, and cold sensation. While functional electrical stimulation (FES) has been studied for motor recovery, its circulatory effects remain less explored. Neuroprosthetic FES (NP-FES), which delivers stimulation synchronized with voluntary electromyography (EMG) activity, may provide additional vascular benefits. This feasibility study aimed to evaluate whether NP-FES can induce observable changes in peripheral circulation in a day rehabilitation setting and whether response timelines differ between severe and mild hemiplegia.

Methods

Sixteen chronic stroke patients (eight severe, Brunnstrom ≤2; 8 mild, ≥5) participated in a randomized crossover trial. Each participant underwent four weeks of NP-FES and four weeks of sensory stimulation, separated by a two-week washout. Estimated blood flow (Q) was modeled from venous vessel width (VVW), skin surface temperature (Skin_Temp), and estimated hemoglobin (Hb) value. Assessments were performed pre-, post-, and 3 minutes post-stimulation at baseline, week 2, and week 4. Analyses included paired t-tests with Bonferroni correction and Bayes factors.

Results

No carryover effects were observed. In the severe group, NP-FES significantly increased Q at week 4, accompanied by an increased contribution of venous vessel width. In the mild group, sensory stimulation produced significant improvements as early as week 2. Subjective improvements, including enhanced limb awareness, initiation of use, and, in some cases, reduced cold sensation, were reported by 50% of patients with severe cases and 75% of mild cases.

Conclusions

Peripheral circulatory responses to NP-FES can be feasibly detected in clinical rehabilitation practice. The distinct timelines observed-four weeks for severe cases and two weeks for mild cases-suggest severity-specific expectations for circulation-based outcomes. These findings highlight feasibility rather than definitive efficacy and provide direction for future randomized controlled trials.

## Introduction

Unfortunately, interest in the physical function of chronic hemiplegia is not high among both patients and medical professionals. The situation is similar not only in developed countries where life expectancy is increasing, but also in countries where medical care is still in its infancy. However, the increase in the number of elderly and disabled people is an important issue that is directly related to the increase in the population requiring long-term care. In Japan, where life expectancy has been extended, a vexing issue is the decline in the maintenance of activities of daily living (ADL) and health-related quality of life (HR-QOL) during the chronic phase of stroke, caused by the loss of motor function and peripheral circulation in the paralyzed limb [1].

In fact, Akbarfahimi et al. found a significant positive correlation between upper extremity function and ADL independence and social participation in chronic stroke patients [2], and Alsubiheen et al. reported that task-oriented ADL training significantly improved upper extremity function, ADL, and quality of life [3]. Among others, patients with severe hemiplegia often report extremely infrequent use of the paralyzed limb, decreased peripheral circulation, and complaints of coldness [4], and prolonged sensory changes and functional limitations immobilize the perception of "inability to move," leading to progressive loss of motor imagery and disuse. Many people exhibit a vicious cycle. This phenomenon is also known by the concepts of "learned nonuse" and "collapse of motor imagery," and it has been reported that long-term nonuse reduces self-efficacy and motivation to exercise, and chronic motor restriction persists without drawing on the potential resilience of physical function [5].

In people with hemiplegia, the perception of "immobility" has become fixed as a countermeasure to this perception, and systematic reviews and meta-analyses have shown that treatment with functional electrical stimulation (FES) is effective in improving motor function and ADLs [6,7]. However, because conventional FES mechanically stimulates at preset timing, it is not synchronized with the patient's exercise intention, and the patient's motivation to actively participate in the treatment is easily impaired. Furthermore, it has been pointed out that the difficulty in predicting the timing of stimulation can lead to anxiety and fear, which can be an obstacle to continued treatment. In fact, as patient needs these technologies, especially from patients with severe paralysis, we also experience many earnest complaints of "when will I see the effects of the treatment?" This uncertainty often leads to premature discontinuation of treatment by both patients and therapists, particularly when observable improvements are not evident within the first few sessions.

Identifying a typical time frame for the onset of effects would provide therapists with evidence-based guidance to support continued engagement. In order to solve the problem of when the effect will become apparent, we focused on Neuroprosthetic Functional Electrical Stimulation (NP-FES ), which induces joint movements synchronized with the patient's motor intention by triggering myoelectric signals of the residual muscle and sending electrical stimulation to the paralyzed side by the patient himself/herself [8]. Unlike conventional FES, NP-FES induces joint movements in synchrony with the patient's motor intentions, thereby promoting active participation in treatment [8]. In a similar approach, Knutson et al. developed CC-FES (Contralaterally Controlled FES), which stimulates the paralyzed side in conjunction with the movements of the nonparalyzed hand, and reported significant improvements in finger extension in chronic and subacute stroke patients after six weeks of training [9].

We have reported that repetitive maximal joint range of motion on the paralyzed side can be achieved under 35 Hz and 70 Hz NP-FES conditions in chronically ill patients more than one year after onset [8], but "when treatment is effective," especially in severely paralyzed cases, remains unclear.

The question of when a treatment technique for hemiplegic patients with FES is effective requires a definition of effectiveness. Since the critical period of brain plasticity has been exceeded, one possible indicator is not the recovery of paralysis itself, but the improvement of the circulatory dynamics of the hand, which has become a disused limb because the patient's perception of "immobility" has become fixed. Edema of the paralyzed limb is frequently observed in elderly patients after stroke [10]. This condition is primarily attributed to immobility and reduced muscle pump activity, leading to impaired lymphatic and venous return. Neuromuscular electrical stimulation (NMES), including FES, has been reported to reduce edema through induced muscle contractions acting as a mechanical pump [11]. Therefore, electrically elicited muscle activation may provide a physiological mechanism by which FES contributes to improved peripheral circulation. In addition, cold sensation caused by circulatory disturbances is a distressing symptom that reduces patients' quality of life, and treatment with FES may also contribute to the reduction of these symptoms.

The evaluation of peripheral circulation is challenged by substantial temporal variability, which limits the reproducibility and sensitivity of single or short‑term measurements using conventional non‑invasive methods [12]. At the microcirculatory level, fluctuations due to vasomotion and environmental factors are particularly pronounced, underscoring the need to integrate and model multiple physiological indices for robust assessment. In this context, wearable multisensor systems combining pulse-wave, skin-color, and temperature measurements, such as those developed by Tomioka et al. [13], have shown promising accuracy in estimating tissue perfusion. This study builds on that concept by modeling estimated peripheral blood flow (Q) from venous vessel width (VVW), skin surface temperature(Skin_Temp), and estimated hemoglobin (Hb) value, to pinpoint when NP‑FES effects emerge and delineate the temporal contributions of VVW and temperature to circulation dynamics. The present study aimed to evaluate whether changes in peripheral circulation induced by NP-FES can be feasibly implemented and observed in a day rehabilitation setting.

Study objectives

Primary objective: To test whether the time course of peripheral circulatory responses-operationalized as estimated blood flow (Q)-to NP-FES versus sensory-level stimulation differs by severity (BRS ≤2 vs ≥5) in chronic stroke, with Week 4 ΔQ3 (3-min Post − Pre) prespecified as the primary endpoint. Secondary objective: To explore perceived changes in awareness and functional use of the affected limb.

## Materials and methods

Participants

Participants were recruited from four outpatient rehabilitation centers. Inclusion criteria were: (1) stroke onset more than one year prior, (2) participation in day rehabilitation at least twice per week, and (3) self-reported difficulty with finger movement. Exclusion criteria included severe joint contractures in the metacarpophalangeal (MP), proximal interphalangeal (PIP), or distal interphalangeal (DIP) joints or passive range of motion (ROM) of ≤ −70° in wrist extension. The study protocol was approved by the Research Ethics Committee of Gunma Paz University (Approval No. PAZ-21-17). Written informed consent was obtained from all participants prior to enrollment. The trial registration was completed with the University Hospital Medical Information Network Center (UMIN) Clinical Trials Registry (Registration ID: UMIN000058828).

A total of 195 individuals were screened across four participating facilities. Of these, 22 met the inclusion criteria and were enrolled in the study. During the intervention period, four participants withdrew for health-related reasons: worsening heart failure (n = 1), COVID-19 infection (n = 1), scheduled surgery (n = 1), and conflicting medical appointments (n = 1). After excluding participants with moderate paralysis (Brunnstrom Recovery Stage 3-4; n = 2), 16 participants completed all intervention phases. The final analysis included 8 participants with severe paralysis (Brunnstrom Stage ≤ 2) and 8 with mild paralysis (Stage ≥ 5).

Study design

This was a randomized, open-label crossover study involving 22 participants who underwent both conditions: (1) neuroprosthetic functional electrical stimulation (NP-FES) with joint motion, and (2) sensory electrical stimulation without joint motion. Each participant served as their own control with random allocation of the intervention order. Sensory stimulation was intentionally selected as an active comparator, using stimulation intensities below the motor threshold to prevent joint motion. This choice was driven by clinical and ethical considerations, as a no-stimulation placebo may diminish participant engagement and introduce dropout bias.

To reduce the risk of information sharing bias among participants, intervention sequences were assigned such that participants from the same facility received the same sequence while participants from different facilities were assigned to opposite sequences. This approach ensured standardized conditions while minimizing treatment allocation recognition through peer interactions. Note that a two-week washout period was established to control for carryover effects in the crossover design. Since the primary outcome of this study was the acute blood flow response immediately after EMS, we determined that the two-week time period setting was appropriate in eliminating short-term effects, based on reports that circulatory responses to neuromuscular electrical stimulation typically decay over 24-72 hours [14].

Comparator rationale

Sensory-level electrical stimulation (below motor threshold; no visible joint motion) was chosen as an active comparator rather than a sham to maintain participant engagement and minimize dropout bias while preserving blinding to treatment expectations at the facility level.

Intervention protocol

In the NP-FES condition, electrical stimulation was applied at an intensity sufficient to induce wrist extension within tolerable limits. Stimulation was triggered by myoelectric signals from the non-paretic side to synchronize with voluntary effort, with 2-second extension and 2-second relaxation cycles for 60 repetitions (total duration per session: 4 minutes). In the sensory stimulation condition, subthreshold stimulation was delivered without eliciting visible joint motion. The same duty cycle and total duration (4 minutes per session) were used. Intensity was titrated each session to remain below the motor threshold.

Stimulation was delivered using a Human-Human Interface (Backyard Brains, USA) and a TENS 3000 device (Roscoe Medical, USA). Electrodes (5 cm × 5 cm) were placed over the extensor digitorum muscles with a 1.2-inch inter-electrode distance. The stimulation parameters were set at 70 Hz frequency and 120 μs pulse width [8]. Each participant received two sessions per week for four weeks, with a two-week washout period between interventions.

Randomization and allocation

A computer-generated 1:1 sequence was produced in Microsoft Excel using the RAND () function. To minimize information sharing within sites, assignments were clustered by facility within each enrollment wave. In a given wave, one facility of a paired set was randomized to NP-FES→sensory and the other to sensory→NP-FES. For subsequent enrollment waves, facility-order assignment was re-randomized de novo, so that the same facility could receive either sequence across different waves. After eligibility confirmation, a site coordinator holding the allocation list scheduled participants accordingly and informed the treating therapist at the time of scheduling. Because the assignment was prepared in facility-level batches prior to scheduling, full allocation concealment before scheduling was not feasible (open-label design); we acknowledge this as a limitation. Primary physiological outcomes were device-based, which helps mitigate expectation bias.

Measurements

Estimated blood flow (Q) was calculated from Venous vessel width (VVW), skin surface temperature (Skin_Temp), and estimated hemoglobin (Hb) value. Assessments were performed at three time points relative to stimulation: before, immediately after, and three minutes post-stimulation. These time points were also assessed at baseline, week 2, and week 4 to capture the time course of circulatory changes.

These parameters were obtained using the ASTRIM FIT (Sysmex, Japan), a noninvasive near-infrared spectroscopy device originally designed for hemoglobin estimation and validated for this purpose [15] . Per manufacturer guidance, the middle finger of the paretic hand was placed in the sensor cradle, which simultaneously and automatically recorded VVW, Skin_Temp (on-board thermistor), and estimated Hb. Thus, no separate ultrasound or external thermometry was used. Model parameters for Q estimation were determined using maximum likelihood estimation (MLE), a widely accepted statistical technique for parameter estimation in probabilistic models [16].

Outcomes and time-contrast definitions. The primary physiologic endpoint was the within-participant change in estimated blood flow (Q) at three prespecified time-contrasts: (a) ΔQ1 (Immediate): Post-Pre; (b) ΔQ2 (Sustained): 3-min Post-Post; (c) ΔQ3 (Overall): 3-min Post-Pre. These contrasts were computed at Baseline, Week 2, and Week 4 for each treatment condition (NP-FES vs sensory).

Environmental control

All assessments were conducted in the same HVAC-controlled room under stable conditions (target temperature, 23-25°C; relative humidity, 40-60%). Before measurement, participants sat quietly for at least 20 minutes to acclimate. For each participant, the same device and the same operator were used across sessions. The device was warmed up and calibrated before each session in accordance with the manufacturer’s guidance. Baseline, Post, and 3-minute Post measurements were obtained without changing posture or limb position. Room temperature and humidity were recorded at each session.

Subjective assessments

Semi-structured interviews were conducted following each intervention phase to capture participants’ subjective experiences. Participants were asked open-ended questions about perceived changes in limb function, willingness to use the affected limb, and any other relevant experiences. Although no standardized questionnaires were used, such qualitative interviews are widely regarded as appropriate for capturing nuanced, individualized perspectives in rehabilitation research [17].

Interview guide and coding

Semi-structured interviews used a brief guide with exemplar prompts: (1) “Since the last session, have you noticed any change in hand/finger function or use?”, (2) “Any change in cold sensation or comfort in the paretic hand?”, (3) “Any change in daily activities or willingness to use the affected hand?”. Responses were categorized by episode (e.g., assisted-hand use initiation/establishment, change in perception of ‘immobility’, ancillary symptoms such as pain/coldness) and tabulated by severity × intervention. No inferential statistics were applied to qualitative data.

Statistical analysis

The primary analysis used paired t-tests (with Bayes factors) to evaluate within-participant changes in Q (Post-Pre, 3-min Post-Post, 3-min Post-Pre) within each severity×intervention cell. Between-order comparisons (carryover/order) were tested separately as described below. Participants were classified by severity of paralysis into severe (Brunnstrom Recovery Stage ≤ 2) and mild (Stage ≥ 5) categories [18]. Intervention effects were analyzed separately for each of the four groups formed by combining severity and intervention type: (a) Severe paralysis × NP-FES, (b) Severe paralysis × Sensory stimulation, (c) Mild paralysis × NP-FES, (d) Mild paralysis × Sensory stimulation, (e) Changes in Q were assessed across three time intervals: (1) Immediate effect: post-stimulation minus pre-stimulation, (2) Sustained effect: 3 minutes post-stimulation minus immediately post-stimulation, and (c) Overall effect: 3 minutes post-stimulation minus pre-stimulation. As Q values were normally distributed, paired t-tests with Bonferroni correction were used for group comparisons.

To assess potential carryover effects in the crossover design, independent-sample t-tests were conducted to compare the effects of intervention order (NP-FES → Sensory vs. Sensory → NP-FES) on estimated blood flow (Q). Analyses were performed across three time intervals (Post-Pre, 3-min Post-Post, and 3-min Post-Pre). Effect sizes were calculated using Hedges’ g.

Severity-stratified analyses. Upper-limb motor severity was classified by the Brunnstrom Recovery Stage (BRS). The primary between-severity comparisons were prespecified to contrast severe (BRS ≤2) versus mild (BRS ≥5). Because we anticipated very low accrual and clinical heterogeneity in the moderate subgroup (BRS 3-4), participants in this category were not included in the primary between-severity analysis. Their data were retained and summarized descriptively and in sensitivity analyses (Appendix 1, 2) to verify that their inclusion would not change the study conclusions.

Carryover/order Check

Potential carryover due to intervention order was evaluated with a repeated-measures general linear model (GLM) with within-subject factor Treatment (NP-FES vs sensory) and between-subject factor Sequence (NP-FES→sensory vs sensory→NP-FES). The primary endpoint for this check was Week 4 ΔQ3 (3-min Post-Pre); robustness analyses were run for Week 4 ΔQ1/ΔQ2 and Week 2 ΔQ1-ΔQ3 (Appendix 3: S3).

Software and Missing Data Handling

Frequentist analyses were conducted in IBM SPSS Statistics version 29.0 (IBM Corp., Armonk, NY); Bayes factors were computed in JASP version 0.19.3 (www.jasp-stats.org). Dropouts were excluded from analysis; within completers, tests were run on a listwise-complete basis for the variables involved in each analysis. No imputation was performed. Bayesian analysis used a default zero-centered Cauchy prior for the standardized effect size (r = 0.707). The Bayes factor is reported as BF₁₀ based on the two-sided alternative hypothesis.

Model Selection and Supplemental Analyses

Four candidate regression models, including different combinations of VVW, Hb, and skin temperature, were compared using Akaike Information Criterion (AIC), Bayesian Information Criterion (BIC), and R² to determine the best-fit model for estimating Q. The optimal model (including VVW and skin temperature) was subsequently used for primary analyses. Additionally, exploratory analyses were conducted by creating six regression models for each severity group at baseline, week 2, and week 4 to examine changes in circulatory dynamics over time via standardized partial regression coefficients (β) for VVW and skin temperature.

Sample Size Consideration

Sample size was estimated based on an expected effect size of 0.5, 80% power, and α = 0.05. Ultimately, 16 participants (n = 8 per severity group) completed the study. Complementary Bayesian paired t-tests were performed to assess reproducibility and the strength of observed effects using Bayes Factors [19]. We note explicitly that this was a feasibility study; the sample-size calculation was used to target a plausible effect (d = 0.5) rather than to power a definitive efficacy trial.

## Results

Subject characteristics

Twenty-two participants were scheduled to complete both the NP-FES and sensory electrical stimulation conditions, but four dropped out during the implementation period due to hospitalization or illness. The reasons for dropouts were worsening heart failure, COVID-19 infection, decision to undergo surgery during regular visits, and scheduled hospital visits on the day of measurement. Eighteen participants completed both treatment periods. Of these, two were classified as moderate paresis (BRS 3-4). As prespecified in the analysis plan, they were not included in the primary between-severity comparison (severe (BRS ≤2) vs mild (BRS ≥5)). Their complete trajectories and within-person changes are provided in Appendices 1, 2 (Tables S1, S2, Figures S1-1, S1-2). The final primary-analysis sample, therefore, comprised 16 participants (severe n=8; mild n=8) (Table 1). Each participant was prescribed eight sessions of electrical stimulation, and all sessions were performed by the therapist in charge in a day rehabilitation facility. The execution rate (mean ± SD) of the prescribed sessions was 91.7 ± 5.4% in the NP-FES group and 86.8 ± 6.2% in the sensory electrical stimulation group. Factors that prevented the implementation were: absence due to the user's physical condition or personal reasons, due to sudden staff absences, and unexpected facility holidays.

**Table 1:** Characteristics of the study subjects Values are presented as N (number of participants), % (percentage), and mean ± standard deviation (SD), as appropriate. Between-group comparisons were conducted using independent-sample t tests for continuous variables and Fisher’s exact test for categorical variables. Two-sided p-values were reported, and statistical significance was defined as p < 0.05. Br.stage_Hand = Brunnstrom Recovery Stage of hand function; FMA-UL = Fugl-Meyer Assessment – Upper Limb; TIS = Trunk Impairment Scale.

	Total ( N =16，100%)	Br.stage_Hand≤Ⅱ (N=8，50%)	Br.stage_Hand≥Ⅴ ( N =8，50%)	Test statistic	P-value
Age(years), mean ± SD	76.8 ± 6.8	72.5 ± 6.5	81.1 ± 4.0	t(14) = -3.22	0.006*
Sex,male n(%)	8 (50.0%)	4 (50.0%)	4 (50.0%)	-	1.000
Height (cm), mean ± SD	158.3 ± 9.0	158.7 ± 11.5	158.0 ± 6.4	t(14) = 0.14	0.889
Weight (kg), mean ± SD	57.4 ± 10.7	59.3 ± 11.5	55.5 ± 10.1	t(14) = 0.71	0.492
BMI, mean ± SD	22.9 ± 3.7	23.7 ± 4.6	22.1 ± 2.7	t(14) = 0.88	0.394
Time since onset (years), mean ± SD	9.4 ± 3.7	9.0 ± 3.9	9.7 ± 8.1	t(13) = -0.20	0.844
Affected side (Right / Left), n (%)	7 / 9 (43.8%/56.2%)	4 / 4 (50.0%/50.0%)	3 / 5 (37.5%/62.5%)	-	1.000
FMA-UL, mean ± SD	34.4 ± 19.7	18.4 ± 8.3	50.4 ± 13.1	t(14) = -5.82	<0.001*
TIS, mean ± SD	13.3 ± 5.2	12.0 ± 4.0	14.5 ± 6.3	t(14) = -0.95	0.357
Intervention order (A→B / B→A), n (%)	8 / 8 (50.0%/50.0%)	5 / 3 (62.5%/37.5%)	6 / 2 (75.0%/25.0%)	-	1.000

Fitness of each model

The following four models were compared to predict changes in estimated blood flow Q (Table 2).

**Table 2:** Comparison of goodness-of-fit indices in predictive models for estimated blood flow (Q) The models were developed to estimate blood flow (Q) based on maximum likelihood estimation (MLE). Values are presented as Akaike Information Criterion (AIC), Bayesian Information Criterion (BIC), and coefficient of determination (R²). Lower AIC and BIC values indicate better model fit, while higher R² values indicate greater predictive accuracy. The Full model included all three predictors (VVW, Hb, and Skin_Temp) and showed R² = 1.000, suggesting potential overfitting; it was therefore excluded from further consideration. The VVW & Skin_Temp model, which showed the best balance among AIC, BIC, and R², was adopted as the primary analytical model in this study. VVW = venous vessel width; Hb = estimated hemoglobin value; Skin_Temp = skin surface temperature.

Model	AIC	BIC	R^2^
Full(VVW,Hb,Skin_Temp)	-1914.91	-1908.58	1.000
VVW&Hb	290.64	295.40	0.170
VVW&Skin_Temp	164.17	168.92	0.975
Hb&Skin_Temp	218.27	223.02	0.889

The Full model (VVW, Hb, Skin_Temp) was excluded due to concerns about overfitting with R²=1.000, and the VVW & Skin_Temp model with low AIC and BIC and high R² was adopted as the optimal model.

Vascular function (change in estimated blood flow Q)

In the analysis of estimated blood flow Q using the VVW & Skin_Temp model, the following significant changes were identified. To evaluate potential carryover effects in the crossover design, independent-sample t-tests were conducted to compare changes in estimated blood flow (Q) between groups that received NP-FES first versus those receiving sensory stimulation first. Analyses were performed across three time intervals: Post-Pre, 3-min Post-Post, and 3-min Post-Pre. As shown in Table 3, no significant differences were observed in any interval (all p > 0.29), and effect sizes (Hedges’ g) ranged from -0.49 to 0.10. These findings support the absence of a carryover effect and validate the 2-week washout period (Table 3).

**Table 3:** Effect of intervention order on estimated blood flow (Q) in the crossover design Values represent changes in estimated blood flow (Q) at each timepoint. Comparison was conducted using independent-samples t-tests based on intervention order (Movement→Sensory vs. Sensory→Movement). Test statistics (t-values and degrees of freedom) and effect sizes (Hedges’ g) are provided. No significant differences were observed between order groups, suggesting no evidence of a carryover effect.

Timepoint	Mean ± SD (Order: Movement→Sensory)	Mean ± SD (Order: Sensory →Movement)	Mean Difference (95% CI)	p-value	t(df)	Effect Size (Hedges' g)
Post - Pre	30.58 ± 67.55	25.12 ± 40.83	5.46 (-59.64 to 70.56)	0.854	t(9.33) = 0.189	0.096
3min - Post	-2.70 ± 30.43	20.43 ± 53.56	-23.13 (-69.00 to 22.74)	0.296	t(13.03) = −1.089	-0.485
3min - Pre	27.88 ± 56.52	45.55 ± 74.96	-17.67 (-88.18 to 52.84)	0.599	t(13.998) = −0.538	-0.247

In the severe × NP-FES group at week 4, ΔQ3(3 min Post - Pre; Overall effect) showed a mean change of 6.98 (95% confidence interval [1.45, 12.50]), p = 0.02; however, the Bayes factor was BF₁₀ = 0.27 (< 1/3), which favors the null (H₀) and therefore indicates limited Bayesian evidence despite the frequentist significance.

Conversely, in the mild × sensory group at week 2, ΔQ1(Post - Pre; Immediate effect) and ΔQ2 (3 min Post - Post; Sustained effect) showed the mean change of 28.95, 95% confidence interval [6.61, 51.28] and 22.01, 95% confidence interval [4.97, 39.05], p = 0.018; while BF₁₀ = 0.24 and 0.25 (both < 1/3) likewise favor H₀, again indicating limited Bayesian evidence despite frequentist significance. (Table 4)

The following table (Table 4) shows the changes (mean ± SD) at baseline, week 2, and week 4 in the four groups of "severe/mild paralysis× with/without intervention." Severity of paralysis was defined as severe for BRS hand ≤2 and mild for ≥5. ‘With intervention’ denotes NP-FES; ‘without intervention’ denotes sensory electrical stimulation (subthreshold). Statistically significant within-group change at week 4 in the severe × with-intervention group (p = 0.02, Bonferroni-corrected).

**Table 4:** Changes in estimated blood flow (ΔQ) by combination of paralysis severity and intervention Values are presented as mean ± standard deviation (SD), t-values with degrees of freedom [t(df)], p-values, 95% confidence intervals (CI), Bayes factors (BF₁₀), Bayesian p-values, and 95% credible intervals. An asterisk (*) indicates statistical significance after Bonferroni correction (p < 0.05). ΔQ1 = Post − Pre (Immediate effect); ΔQ2 = 3 min Post − Post (Sustained effect); ΔQ3 = 3 min Post − Pre (Overall effect). Bayes factor (BF₁₀) interpretation: > 3 supports H₁; 1/3–3 inconclusive; < 1/3 supports H₀. All Bayes factors are reported as BF₁₀ (two-sided).

Group	Time Point	ΔQ	Effect Type (Formula)	Mean ± SD	t(df)	p-value	95% CI (Lower, Upper)	BF	Bayesian p-value	95% Credible Interval (Lower, Upper)
Severe paresis ×With intervention(N=8，50%)	Baseline	ΔQ1	Immediate	-3.93 ± 24.91	t(7)=-.447	0.67	-24.76, 16.89	3.56	0.67	-30.72, 22.86
		ΔQ2	Sustained	4.92 ± 29.32	t(7)=.475	0.65	-19.59, 29.43	3.52	0.65	-26.61, 36.45
		ΔQ3	Overall	8.85 ± 17.13	t(7)=1.462	0.19	-5.47, 23.17	1.61	0.19	-9.57, 27.27
	Week 2	ΔQ1	Immediate	27.92 ± 37.51	t(7)=2.105	0.07	-3.44, 59.28	0.78	0.07	-12.41, 68.26
		ΔQ2	Sustained	19.34 ± 40.42	t(7)=1.353	0.22	-14.45, 53.13	1.81	0.22	-24.13, 62.80
		ΔQ3	Overall	-8.59 ± 24.16	t(7)=-1.005	0.35	-28.78, 11.61	2.49	0.35	-34.56, 17.39
	Week 4	ΔQ1	Immediate	12.75 ± 28.26	t(7)=1.277	0.24	-10.87, 36.38	1.95	0.24	-17.63, 43.14
		ΔQ2	Sustained	19.73 ± 28.14	t(7)=1.984	0.09	-3.79, 43.25	0.90	0.09	-10.52, 49.99
		ΔQ3	Overall	6.98 ± 6.61	t(7)=2.987	0.02*	1.45, 12.50	0.27	0.02*	-0.13, 14.08
Severe paresis ×Without intervention( N =8，50%)	Baseline	ΔQ1	Immediate	17.73 ± 24.84	t(7)=2.019	0.08	-3.04, 38.50	0.86	0.08	-8.99, 44.44
		ΔQ2	Sustained	21.64 ± 29.33	t(7)=2.087	0.08	-2.88, 46.16	0.79	0.08	-9.90, 53.18
		ΔQ3	Overall	3.91 ± 21.46	t(7)=.515	0.62	-14.03, 21.85	3.45	0.62	-19.17, 26.99
	Week 2	ΔQ1	Immediate	7.28 ± 27.69	t(7)=.744	0.48	-15.87, 30.43	3.03	0.48	-22.49, 37.06
		ΔQ2	Sustained	8.29 ± 23.72	t(7)=.988	0.36	-11.54, 28.13	2.53	0.36	-17.22, 33.80
		ΔQ3	Overall	1.01 ± 16.94	t(7)=.169	0.87	-13.15, 15.17	3.86	0.87	-17.21, 19.22
	Week 4	ΔQ1	Immediate	9.64 ± 40.94	t(7)=.666	0.53	-24.58, 43.86	3.18	0.53	-34.38, 53.66
		ΔQ2	Sustained	-3.44 ± 26.10	t(7)=-.373	0.72	-25.27, 18.38	3.66	0.72	-31.52, 24.63
		ΔQ3	Overall	-13.09 ± 31.45	t(7)=-1.177	0.28	-39.38, 13.21	2.14	0.28	-46.91, 20.74
Mild paresis ×With intervention( N =8，50%)	Baseline	ΔQ1	Immediate	6.20 ± 31.98	t(7)=.548	0.60	-20.54, 32.94	3.40	0.60	-28.19, 40.59
		ΔQ2	Sustained	-0.68 ± 34.25	t(7)=-.056	0.96	-29.31, 27.95	3.90	0.96	-37.51, 36.15
		ΔQ3	Overall	-6.88 ± 22.13	t(7)=-.879	0.41	-25.39, 11.62	2.76	0.41	-30.68, 16.92
	Week 2	ΔQ1	Immediate	8.46 ± 36.72	t(7)=.652	0.54	-22.24, 39.16	3.21	0.54	-31.03, 47.95
		ΔQ2	Sustained	2.31 ± 27.20	t(7)=.240	0.82	-20.44, 25.05	3.80	0.82	-26.95, 31.56
		ΔQ3	Overall	-6.16 ± 11.56	t(7)=-1.506	0.18	-15.82, 3.51	1.54	0.18	-18.59, 6.27
	Week 4	ΔQ1	Immediate	11.04 ± 35.74	t(7)=.873	0.41	-18.84, 40.92	2.77	0.41	-27.40, 49.47
		ΔQ2	Sustained	24.78 ± 54.60	t(7)=1.283	0.24	-20.87, 70.42	1.93	0.24	-33.94, 83.49
		ΔQ3	Overall	13.74 ± 29.06	t(7)=1.337	0.22	-10.56, 38.04	1.83	0.22	-17.52, 44.99
Mild paresis ×Without intervention( N =8，50%)	Baseline	ΔQ1	Immediate	22.31 ± 63.75	t(7)=.990	0.36	-30.98, 75.60	2.52	0.36	-46.24, 90.86
		ΔQ2	Sustained	14.96 ± 71.81	t(7)=.589	0.57	-45.08, 74.99	3.33	0.57	-62.27, 92.18
		ΔQ3	Overall	-7.35 ± 28.63	t(7)=-.726	0.49	-31.29, 16.59	3.07	0.49	-38.15, 23.44
	Week 2	ΔQ1	Immediate	28.95 ± 26.72	t(7)=3.065	0.018*	6.61, 51.28	0.24	0.018*	0.22, 57.68
		ΔQ2	Sustained	22.01 ± 20.38	t(7)=3.055	0.018*	4.97, 39.05	0.25	0.018*	0.10, 43.93
		ΔQ3	Overall	-6.94 ± 14.90	t(7)=-1.317	0.23	-19.39, 5.52	1.87	0.23	-22.96, 9.09
	Week 4	ΔQ1	Immediate	21.59 ± 44.18	t(7)=1.382	0.21	-15.35, 58.52	1.75	0.21	-25.92, 69.09
		ΔQ2	Sustained	34.57 ± 52.97	t(7)=1.846	0.11	-9.71, 78.85	1.05	0.11	-22.39, 91.53
		ΔQ3	Overall	12.98 ± 32.45	t(7)=1.131	0.30	-14.15, 40.11	2.23	0.30	-21.91, 47.88

**Figure 1:**
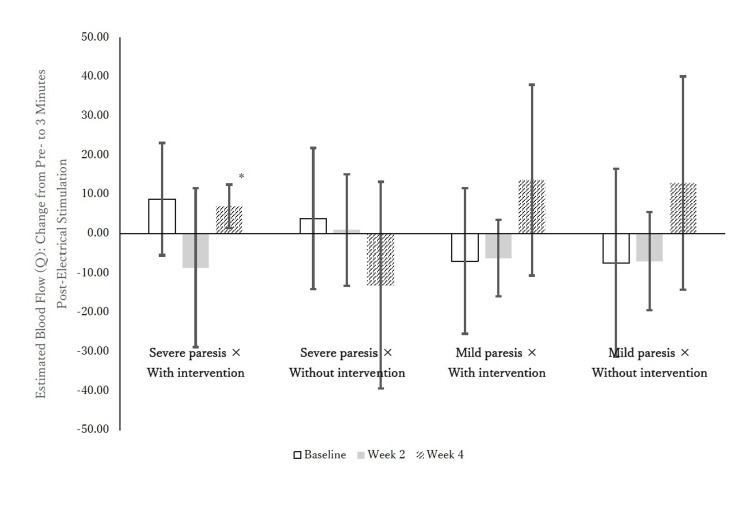
Change in estimated blood flow Q after implementation of electrical stimulation (3-min Post-Pre) Changes in estimated blood flow (Q) after electrical stimulation, calculated as the difference between 3 minutes post-stimulation and pre-stimulation. Values are presented as mean ± standard deviation (SD). Bars represent mean changes in Q at baseline, week 2, and week 4 for each group: (a) Severe paresis with intervention (neuroprosthetic functional electrical stimulation (NP-FES)), (b) Severe paresis without intervention (sensory electrical stimulation), (c) Mild paresis with intervention (NP-FES), (d) Mild paresis without intervention (sensory electrical stimulation), (e) White bars indicate baseline, gray bars indicate week 2, and hatched bars indicate week 4. Error bars represent SD. *Indicates a statistically significant difference from baseline (p < 0.05, Bonferroni corrected). Q = estimated blood flow; NP-FES = neuroprosthetic functional electrical stimulation.

Only the severe × NP-FES group shows a clear week-4 increase in ΔQ3 (3-min Post − Pre) that reaches statistical significance, whereas the other groups exhibit small or variable changes with overlapping dispersion, and the mild × sensory group’s overall ΔQ3 remains near zero despite its week-2 immediate/sustained effects (Figure 1). Figure 1 shows the change in estimated blood flow Q after the implementation of electrical stimulation (after 3 min before implementation). 

Sensitivity analysis, including the moderate subgroup

To verify robustness, we repeated the carryover/order analysis after re-introducing the two participants with moderate paresis (BRS 3-4). There was no Treatment×Sequence interaction at the primary endpoint (Week 4 ΔQ3: F(1,16)=0.288, p=0.599, partial η²=0.018), indicating no evidence of carryover when the moderate cases are included. Details are provided in Appendix 3 (Table S3).

Contribution of VVW and skin surface temperature (supplemental analysis)

As a supplemental analysis, six model equations were created for the initial, two-week, and four-week follow-up for the severe and mild paresis groups, respectively, and standardized partial regression coefficients (β) and correlation coefficients for VVW and skin surface temperature (Skin_Temp) were compared. In the severe paralysis group, the VVW contribution (β = 0.523, correlation = 0.722) increased after 4 weeks from the initial time point and was close to the skin surface temperature (β = 0.720, correlation = 0.864). In particular, the VVW contribution tended to be higher than that of mild paralysis at two and four weeks. In contrast, in the mild paresis group, the contribution of VVW (β = 0.456, correlation = 0.770) remained relatively stable and was a contributing factor to skin surface temperature (β = 0.711, correlation = 0.912) at two and four weeks, the contribution of skin surface temperature remained at a higher level than in the severe paresis group (Table 5).

**Table 5:** Changes in the contribution of VVW and skin surface temperature (results of multiple regression analysis by group and time point) Values are presented as standardized regression coefficients (β) and correlation coefficients for each predictor (VVW, Skin_Temp) in multiple regression models stratified by Brunnstrom stage (Br ≤ 2: severe paresis; Br ≥ 5: mild paresis) and time point. VVW = venous vessel width; Skin_Temp = skin surface temperature; Br = Brunnstrom Recovery Stage.

Group	Time Point	R² (Coefficient of Determination)	Primary Contributing Factor	β（VVW）	β（Skin_Temp）	Correlation (VVW)	Correlation (Skin Temp)
Br≤2	Baseline	1.000	Skin_Temp	0.176	1.022	-0.041	0.985
Br≤2	Week 2	1.000	Skin_Temp	0.268	1.045	-0.038	0.967
Br≤2	Week 4	1.000	Skin_Temp + VVW	0.523	0.72	0.722	0.864
Br≥5	Baseline	1.000	Skin_Temp + VVW	0.328	0.764	0.798	0.966
Br≥5	Week 2	1.000	Skin_Temp + VVW	0.542	0.658	0.794	0.866
Br≥5	Week 4	1.000	Skin_Temp + VVW	0.456	0.711	0.77	0.912

Subjective changes reported after electrical stimulation intervention were categorized and tabulated by severity of paralysis (Brunnstrom Recovery Stage: severe=hand 2 or less, mild=hand 5 or more) and intervention method (NP-FES, sensory electrical stimulation) (Table 6). Four (50.0%) in the severe paresis group and six (75.0%) in the mild paresis group showed a change in their perception of "not being able to move. The most common response was "use of an assistive hand," which was reported by four (50.0%) in the severe paresis group and by five (62.5%) in the mild paresis group. Regarding the use of the assisted hand, "initiation of use" was reported by three (37.5%) in the severe group and two (25.0%) in the mild group, and "establishment of use" was reported by one (12.5%) in the severe group and three (37.5%) in the mild group.

**Table 6:** Classification and frequency of subjective changes observed after electrical stimulation intervention, by severity of paralysis and intervention NP-FES = neuroprosthetic functional electrical stimulation; Sensory stimulation = perceptible electrical stimulation without visible motor response; Brs = Brunnstrom Recovery Stage. “Severe” = Brs ≤ 2; “Mild” = Brs ≥ 5. Each group included 8 participants (n = 8). Values are presented as n (%). Subjective changes were classified and summarized for 8 participants in each group. The number in each cell indicates the number of participants who reported a change, and the percentage in parentheses represents the proportion relative to the total in each group (n = 8).

Subjective Change Category	Severity of Paresis, n (%)	NP-FES (With Intervention), n (%)	Sensory Stimulation (Without Intervention), n (%)	Total, n (%)
Change in Perception of “Inability to Move” (Combined)	Severe(n=8,50%)	3 (37.5%)	1 (12.5%)	4 (50.0%)
	Mild(n=8,50%)	5 (62.5%)	1 (12.5%)	6 (75.0%)
Use of the affected hand (Combined)	Severe	3 (37.5%)	1 (12.5%)	4 (50.0%)
	Mild	4 (50.0%)	1 (12.5%)	5 (62.5%)
Initiation of use	Severe	2 (25.0%)	1 (12.5%)	3 (37.5%)
	Mild	2 (25.0%)	0 (0.0%)	2 (25.0%)
Habitual use	Severe	1 (12.5%)	0 (0.0%)	1 (12.5%)
	Mild	2 (25.0%)	1 (12.5%)	3 (37.5%)
Motivation to use / Sense of self-efficacy	Severe	0 (0.0%)	0 (0.0%)	0 (0.0%)
	Mild	1 (12.5%)	0 (0.0%)	1 (12.5%)
Supplementary Clinical Changes (Combined)	Severe(n=8,50%)	3 (37.5%)	0 (0.0%)	3 (37.5%)
	Mild(n=8,50%)	0 (0.0%)	0 (0.0%)	0 (0.0%)
Pain reduction	Severe	1 (12.5%)	0 (0.0%)	1 (12.5%)
	Mild	0 (0.0%)	0 (0.0%)	0 (0.0%)
Improvement in cold sensation	Severe	1 (12.5%)	0 (0.0%)	1 (12.5%)
	Mild	0 (0.0%)	0 (0.0%)	0 (0.0%)
Changes in sensory perception	Severe	1 (12.5%)	0 (0.0%)	1 (12.5%)
	Mild	0 (0.0%)	0 (0.0%)	0 (0.0%)

A change in willingness to use and self-efficacy was identified by one (12.5%) in the mild group. Regarding ancillary clinical changes, three (37.5%) of the severe group reported some changes, while none were reported by the mild group. Specifically, one (12.5%) each reported a reduction in pain, an improvement in cold sensation, and a change in sensory perception. None of the symptom relief was reported in the sensory electrical stimulation group. In summary, significant changes in estimated blood flow (Q) were observed at week 4 in severe cases with NP-FES, and at week 2 in mild cases, even under sensory stimulation.

## Discussion

This study investigated the feasibility of assessing peripheral circulatory responses to neuroprosthetic functional electrical stimulation (NP-FES) in chronic stroke patients. Using an integrated hemodynamic model based on venous vessel width and skin temperature, we demonstrated that time-dependent changes in estimated blood flow (Q) can be detected in a day rehabilitation setting. Importantly, the observed patterns varied by severity of hemiplegia, highlighting the clinical relevance of tailoring stimulation protocols.

In the severe hemiplegia group, significant increases in Q were observed only after four weeks of NP-FES intervention. This delayed response may reflect the time required for repeated muscle pump activation to induce vascular remodeling and shear stress-mediated nitric oxide release [20-23]. Prior exercise and NMES studies suggest that vascular adaptations typically emerge after several weeks of consistent stimulation [24,25], consistent with our findings. Thus, for patients with severe paralysis, sustained intervention appears essential before measurable circulatory improvements can be expected.

In contrast, the mildly affected group exhibited earlier improvements, with significant increases in Q already evident at week two under sensory stimulation. This finding is in line with reports that somatosensory input alone can facilitate motor performance and modulate cortical excitability in chronic stroke [26,27] . Preserved vascular architecture and endothelial function in mild cases [28,29] may enable more rapid responsiveness, even under subthreshold stimulation. Clinically, this suggests that low-burden sensory stimulation may represent a feasible early-phase strategy for patients with mild impairments, whereas longer NP-FES intervention is warranted for severe cases.

Beyond physiological outcomes, subjective reports of improved limb awareness and initiation of use were frequent, particularly in the NP-FES group. These observations may reflect disruption of learned nonuse [5] and reinforcement of sensorimotor feedback loops, consistent with prior behavioral studies. Although qualitative in nature, such findings highlight the potential for NP-FES to influence not only peripheral circulation but also patient motivation and engagement. These qualitative observations were collected without standardized instruments and are therefore preliminary and descriptive; they should be interpreted cautiously and triangulated with validated patient-reported measures in future trials.

The crossover design allowed each participant to serve as their own control. We found no evidence of carryover effects with a two-week washout period, consistent with prior reports that NMES effects dissipate within 72 hours [14]. At the same time, complementary use of Bayes factors provided a nuanced interpretation, highlighting cases where statistical significance and Bayesian evidence diverged. Such contrasts reinforce the need for multifaceted inference in small-sample rehabilitation studies. Clinically, identifying week four as a therapeutic threshold in severe hemiplegia and week two in mild cases provides practical guidance for therapy planning. These timeframes may help therapists manage patient expectations, sustain motivation, and optimize treatment adherence. The evidence also supports sensory stimulation as a viable early-phase intervention in mildly affected patients, while underscoring the necessity of longer NP-FES protocols for severe cases.

Limitations and future directions

Several limitations must be acknowledged. First, the sample size was small (n = 16), limiting generalizability and subgroup analysis. Second, Q values were derived from non-invasive physiological indices and may be influenced by environmental conditions, though these were controlled through climate-regulated facilities and synchronized measurements. Third, subjective outcomes were collected through interviews rather than validated questionnaires, limiting comparability across studies. Despite these limitations, the crossover design and Bayesian analyses strengthen internal validity.

Several limitations should be considered. First, this was an open-label feasibility study conducted with facility clusters, so full allocation concealment prior to scheduling was not feasible, and neither participants nor assessors were blinded; these features may bias subjective outcomes and procedural aspects of measurement. Second, the sample size was small (n=16) and follow-up was short (four weeks per intervention), which limits generalizability, precision of estimates, and inference about durability. Third, the stimulation dose was not fully logged: although the session length was fixed at 4 minutes and repetition counts/motor-response targets were standardized, absolute current (mA) and delivered charge were not recorded, which reduces the reproducibility of the dosing protocol. Fourth, the primary physiologic endpoint (Q) was derived from non-invasive indices (VVW, skin temperature, Hb) that, despite environmental control (HVAC room, acclimation, fixed posture/limb position), may still be sensitive to ambient factors. Fifth, qualitative outcomes were obtained via semi-structured interviews without validated scales, which can reduce reproducibility and introduce interviewer expectancy. Finally, causal interpretation is limited: in a small feasibility crossover, results should be viewed as hypothesis-generating associations, not definitive efficacy.

Future work should comprise larger, prospectively registered, randomized trials with severity stratification, blinded assessment, and complete dose logging (including current and charge). A longer follow-up is needed to test durability. Incorporating validated patient-reported measures and neurophysiological readouts (e.g., fNIRS/EEG/fMRI) will help clarify both peripheral and central mechanisms of NP-FES and strengthen causal inference.

## Conclusions

This feasibility study confirmed that NP-FES was associated with measurable peripheral circulatory responses in elderly post-stroke patients, with distinct time courses depending on the severity of hemiplegia. Severe cases required approximately four weeks of intervention before clear changes were observed, whereas mild cases responded earlier, even under sensory stimulation. These findings do not establish definitive efficacy but demonstrate the implementability of NP-FES as a clinically applicable intervention. The observed patterns offer valuable insights for future large-scale trials aimed at validating its therapeutic potential and optimizing individualized protocols.

## Figures and Tables

**Figure 2:**
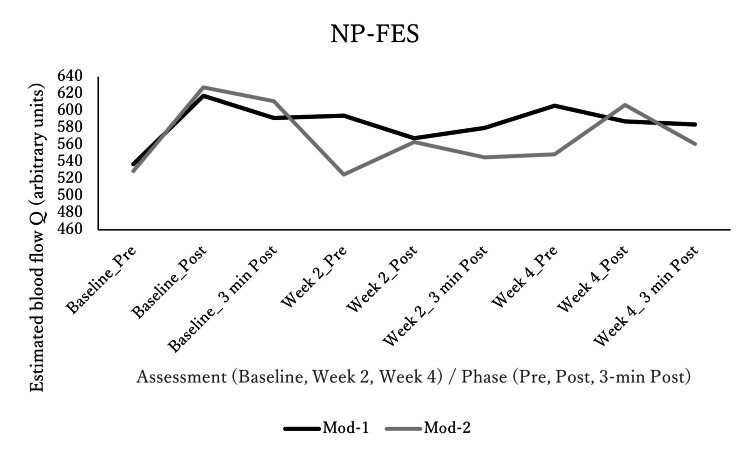
S1-1: NP-FES condition: individual trajectories of estimated blood flow (Q) in the moderate subgroup (BRS 3–4) Lines show within-person trajectories for the two moderate participants (Mod-1, Mod-2). Q (arbitrary units) was model-derived from venous vessel width (VVW) and skin surface temperature (Skin_Temp). Points denote Pre, Post, and 3-min Post at Baseline, Week 2, and Week 4; the same posture and limb position were maintained at each time point. Higher values indicate greater estimated perfusion. These plots are descriptive and are provided to document the full trajectories of the moderate cases. Q, estimated blood flow; NP-FES, neuro-prosthetic functional electrical stimulation; Pre, pre-stimulation; Post, immediately post-stimulation; 3-min Post, 3 minutes after stimulation.

**Figure 3:**
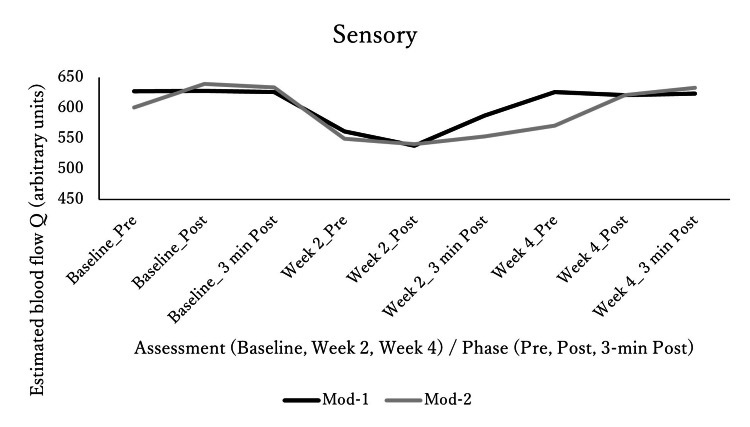
S1-2: Sensory-level stimulation: individual trajectories of estimated blood flow (Q) in the moderate subgroup (BRS 3–4) Lines show within-person trajectories for the two moderate participants (Mod-1, Mod-2). Q (arbitrary units) was model-derived from venous vessel width (VVW) and skin surface temperature (Skin_Temp). Points denote Pre, Post, and 3-min Post at Baseline, Week 2, and Week 4; the same posture and limb position were maintained at each time point. Higher values indicate greater estimated perfusion. These plots are descriptive and are provided to document the full trajectories of the moderate cases. Q, estimated blood flow; Pre, pre-stimulation; Post, immediately post-stimulation; 3-min Post, 3 minutes after stimulation.

## Appendices

Appendix 1. Individual trajectories for two moderate cases

This Appendix 1(Table 7 (S1)) provides individual time courses for the two participants with moderate paresis (BRS 3-4). For each treatment (NP-FES (Figure 2 (S1-1)), sensory stimulation (Figure 3 (S1-2)), estimated blood flow (Q), venous vessel width (VVW), skin temperature (Skin_Temp), and estimated hemoglobin (Hb), the values are shown at Pre-, Post-, and 3-min Post across Baseline, Week 2, and Week 4.

**Table 7:** S1: Individual trajectories for the moderate subgroup (BRS 3–4) Raw measurements for Mod-1 and Mod-2 by Treatment, Assessment, and Phase (Q, Hb, VVW, Skin_Temp). Q is model-derived (arbitrary units); VVW is the device output in cm; Skin_Temp in °C; Hb in g/dL. Measurements were obtained with ASTRIM FIT (Sysmex, Japan) using the middle-finger sensor per manufacturer guidance. Gender (0=male, 1=female); Affiliation (facility identifier); Sequence: NP-FES→Sensory or Sensory→NP-FES; Phase: Pre, Post, 3-min Post.

ID	Br_stage_Fingers	Gender	Affiliation	Sequence	Treatment	Assessment	Phase	Q	Hb	VVW	Skin_Temp
Mod-1	4	0	1	NP-FES⇒Sensory	NP-FES	Baseline	Pre	536.87	14.5	0.58	29.3
Mod-1	4	0	1	NP-FES⇒Sensory	NP-FES	Baseline	Post	617.725	13.9	1.15	33.3
Mod-1	4	0	1	NP-FES⇒Sensory	NP-FES	Baseline	3-min Post	591.59	14.9	1.06	32
Mod-1	4	0	1	NP-FES⇒Sensory	NP-FES	Week 2	Pre	594.04	15.5	1.36	32.1
Mod-1	4	0	1	NP-FES⇒Sensory	NP-FES	Week 2	Post	567.545	13.8	1.03	30.8
Mod-1	4	0	1	NP-FES⇒Sensory	NP-FES	Week 2	3-min Post	579.815	15.7	1.21	31.4
Mod-1	4	0	1	NP-FES⇒Sensory	NP-FES	Week 4	Pre	605.875	15.2	1.25	32.7
Mod-1	4	0	1	NP-FES⇒Sensory	NP-FES	Week 4	Post	587.56	15.7	1.04	31.8
Mod-1	4	0	1	NP-FES⇒Sensory	NP-FES	Week 4	3-min Post	583.68	13.9	1.12	31.6
Mod-1	4	0	1	NP-FES⇒Sensory	Sensory	Baseline	Pre	627.365	15.8	0.91	33.8
Mod-1	4	0	1	NP-FES⇒Sensory	Sensory	Baseline	Post	627.875	14.9	1.25	33.8
Mod-1	4	0	1	NP-FES⇒Sensory	Sensory	Baseline	3-min Post	625.74	15.1	1.16	33.7
Mod-1	4	0	1	NP-FES⇒Sensory	Sensory	Week 2	Pre	561.68	15.1	1.12	30.5
Mod-1	4	0	1	NP-FES⇒Sensory	Sensory	Week 2	Post	537.755	13.7	1.17	29.3
Mod-1	4	0	1	NP-FES⇒Sensory	Sensory	Week 2	3-min Post	587.26	13.9	0.84	31.8
Mod-1	4	0	1	NP-FES⇒Sensory	Sensory	Week 4	Pre	625.74	16.2	1.16	33.7
Mod-1	4	0	1	NP-FES⇒Sensory	Sensory	Week 4	Post	620.87	13.6	0.58	33.5
Mod-1	4	0	1	NP-FES⇒Sensory	Sensory	Week 4	3-min Post	623.755	16.1	1.17	33.6
Mod-2	4	0	1	NP-FES⇒Sensory	NP-FES	Baseline	Pre	528.84	15.1	0.56	28.9
Mod-2	4	0	1	NP-FES⇒Sensory	NP-FES	Baseline	Post	627.215	16	0.81	33.8
Mod-2	4	0	1	NP-FES⇒Sensory	NP-FES	Baseline	3-min Post	611.245	14.6	0.83	33
Mod-2	4	0	1	NP-FES⇒Sensory	NP-FES	Week 2	Pre	525.005	12.9	0.67	28.7
Mod-2	4	0	1	NP-FES⇒Sensory	NP-FES	Week 2	Post	562.945	14.4	0.63	30.6
Mod-2	4	0	1	NP-FES⇒Sensory	NP-FES	Week 2	3-min Post	545.335	14.6	0.89	29.7
Mod-2	4	0	1	NP-FES⇒Sensory	NP-FES	Week 4	Pre	548.915	13.8	0.61	29.9
Mod-2	4	0	1	NP-FES⇒Sensory	NP-FES	Week 4	Post	606.705	16.9	0.47	32.8
Mod-2	4	0	1	NP-FES⇒Sensory	NP-FES	Week 4	3-min Post	560.81	17.1	0.54	30.5
Mod-2	4	0	1	NP-FES⇒Sensory	Sensory	Baseline	Pre	600.6	16.2	0.4	32.5
Mod-2	4	0	1	NP-FES⇒Sensory	Sensory	Baseline	Post	639.02	15.3	0.68	34.4
Mod-2	4	0	1	NP-FES⇒Sensory	Sensory	Baseline	3-min Post	633.755	14.5	1.17	34.1
Mod-2	4	0	1	NP-FES⇒Sensory	Sensory	Week 2	Pre	549.215	1.3	0.81	29.9
Mod-2	4	0	1	NP-FES⇒Sensory	Sensory	Week 2	Post	540.81	20.1	0.54	29.5
Mod-2	4	0	1	NP-FES⇒Sensory	Sensory	Week 2	3-min Post	553.05	14.9	0.7	30.1
Mod-2	4	0	1	NP-FES⇒Sensory	Sensory	Week 4	Pre	570.735	16.6	0.49	31
Mod-2	4	0	1	NP-FES⇒Sensory	Sensory	Week 4	Post	620.855	14.9	0.57	33.5
Mod-2	4	0	1	NP-FES⇒Sensory	Sensory	Week 4	3-min Post	632.915	14.8	0.61	34.1

Appendix 2

Paired within-person changes (ΔQ) for the two moderate cases are summarized for each treatment and time point (Table 8 (S2)). ΔQ1 = Post-Pre (Immediate), ΔQ2 = 3-min Post-Post (Sustained), ΔQ3 = 3-min Post-Pre (Overall). With N=2, estimates are inherently imprecise; results are descriptive.

**Table 8:** S2: Paired with in-person changes in the moderate subgroup Paired differences (Mean ± SD), t(df=1), two-sided p, 95% CI, and BF10 for ΔQ1/ΔQ2/ΔQ3 at Baseline/Week 2/Week 4 under NP-FES and sensory. With N=2, inferential metrics are unstable and should be interpreted cautiously.

Group	Time Point	ΔQ	Effect Type (Formula)	Mean ± SD	t(df)	p-value	95% CI (lower, upper)	BF10
Moderate paresis ×	Baseline	ΔQ1	Immediate	89.62 ± 12.39	t(1)=10.230	0.062	−21.69, 200.92	2.56
With intervention	Baseline	ΔQ2	Sustained	−21.05 ± 7.19	t(1)=−4.142	0.151	−85.63, 43.53	1.62
(N=2)	Baseline	ΔQ3	Overall	68.56 ± 19.58	t(1)=4.953	0.127	−107.32, 244.45	1.8
	Week 2	ΔQ1	Immediate	5.72 ± 45.56	t(1)=0.178	0.888	−403.64, 415.08	0.44
	Week 2	ΔQ2	Sustained	−2.67 ± 21.13	t(1)=−0.179	0.887	−192.50, 187.16	0.44
	Week 2	ΔQ3	Overall	3.05 ± 24.43	t(1)=0.177	0.889	−216.48, 222.58	0.44
	Week 4	ΔQ1	Immediate	19.74 ± 53.81	t(1)=0.519	0.695	−463.77, 503.24	0.5
	Week 4	ΔQ2	Sustained	−24.89 ± 29.71	t(1)=−1.185	0.446	−291.81, 242.04	0.71
	Week 4	ΔQ3	Overall	−5.15 ± 24.11	t(1)=−0.302	0.813	−221.73, 211.43	0.45
Moderate paresis ×	Baseline	ΔQ1	Immediate	19.47 ± 26.81	t(1)=1.027	0.492	−221.38, 260.31	0.65
Without intervention	Baseline	ΔQ2	Sustained	−3.70 ± 2.21	t(1)=−2.364	0.255	−23.59, 16.19	1.13
(N=2)	Baseline	ΔQ3	Overall	15.77 ± 24.59	t(1)=0.907	0.531	−205.20, 236.73	0.61
	Week 2	ΔQ1	Immediate	−16.17 ± 10.97	t(1)=−2.083	0.285	−114.77, 82.44	1.04
	Week 2	ΔQ2	Sustained	30.87 ± 26.35	t(1)=1.657	0.346	−205.88, 267.62	0.88
	Week 2	ΔQ3	Overall	14.71 ± 15.38	t(1)=1.353	0.405	−123.44, 152.86	0.77
	Week 4	ΔQ1	Immediate	22.63 ± 38.88	t(1)=0.823	0.562	−326.73, 371.98	0.58
	Week 4	ΔQ2	Sustained	7.47 ± 6.49	t(1)=1.629	0.351	−50.82, 65.76	0.87
	Week 4	ΔQ3	Overall	30.10 ± 45.37	t(1)=0.938	0.52	−377.55, 437.74	0.62

Appendix 3

Table 9 (S3) shows carryover/order analysis including all three severity groups.

**Table 9:** S3: Carryover/order analysis including all three severity groups Repeated-measures GLM with within-subject factor Treatment (NP-FES vs Sensory), between-subject factor Sequence (NP-FES→Sensory vs Sensory→NP-FES), run on Week-4 ΔQ3 (primary). Provide F, p, partial η² for Treatment, Sequence, and Treatment×Sequence; EMMs by Treatment and by Sequence×Treatment. Add a short sensitivity block for Week-4 ΔQ1/ΔQ2 and Week-2 ΔQ1–ΔQ3. Carryover/order analysis with the moderate subgroup included; all tests non-significant at the primary endpoint and in sensitivity checks.

Effect	F	df1	df2	p-value	partial η²
Treatment (NP-FES vs Sensory)	0.023	1	16	0.88	0.001
Sequence (AB vs BA)	0.35	1	16	0.562	0.021
Treatment × Sequence	0.288	1	16	0.599	0.018
